# Integrated community case management by drug sellers influences appropriate treatment of paediatric febrile illness in South Western Uganda: a quasi-experimental study

**DOI:** 10.1186/s12936-017-2072-9

**Published:** 2017-10-23

**Authors:** Freddy Eric Kitutu, Joan Nakayaga Kalyango, Chrispus Mayora, Katarina Ekholm Selling, Stefan Peterson, Henry Wamani

**Affiliations:** 1 Pharmacy Department, Makerere University College of Health Sciences, Kampala, Uganda; 2 School of Public Health, Makerere University College of Health Sciences, Kampala, Uganda; 3 International Maternal and Child Health Unit, Department of Women’s and Children’s Health, Uppsala University, 751 85 Uppsala, Sweden; 4 Health Section, UNICEF, 3 UN Plaza, New York, NY 10017 USA; 5 Clinical Epidemiology and Biostatistics Unit, School of Medicine, Makerere University College of Health Sciences, Kampala, Uganda; 6 School of Public Health, University of Witwatersrand, 27 St. Andrews Road, Parktown, Johannesburg, 2193 South Africa

**Keywords:** Integrated case management, Drug sellers, Uganda, Febrile illness, Malaria, Pneumonia, Private sector, Diagnostics, Appropriate treatment

## Abstract

**Background:**

Fever case management is a major challenge for improved child health globally, despite existence of cheap and effective child survival health technologies. The integrated Community Case Management (iCCM) intervention of paediatric febrile illnesses though adopted by Uganda’s Ministry of Health to be implemented by community health workers, has not addressed the inaccess to life-saving medicines and diagnostics. Therefore, the iCCM intervention was implemented in private drug shops and evaluated for its effect on appropriate treatment of paediatric fever in a low malaria transmission setting in South Western Uganda.

**Methods:**

From June 2013 to September 2015, the effect of the iCCM intervention on drug seller paediatric fever management and adherence to iCCM guidelines was assessed in a quasi-experimental study in South Western Uganda. A total of 212 care-seeker exit interviews were done before and 285 after in the intervention arm as compared to 216 before and 268 care-seeker interviews at the end of the study period in the comparison arm. The intervention effect was assessed by difference-in-difference analysis of drug seller treatment practices against national treatment recommendations between the intervention and comparison arms. Observed proportions among care-seeker interviews were compared with corresponding proportions from 5795 child visits recorded in patient registries and 49 direct observations of drug seller–care-seeker encounters in intervention drug shops.

**Results:**

The iCCM intervention increased the appropriate treatment of uncomplicated malaria, pneumonia symptoms and non-bloody diarrhoea by 80.2% (95% CI 53.2–107.2), 65.5% (95% CI 51.6–79.4) and 31.4% (95% CI 1.6–61.2), respectively. Within the intervention arm, drug seller scores on appropriate treatment for pneumonia symptoms and diagnostic test use were the same among care-seeker exit interviews and direct observation. A linear trend (negative slope, − 0.009 p value < 0.001) was observed for proportions of child cases prescribed any antimicrobial medicine in the intervention arm drug shops.

**Conclusions:**

The iCCM intervention improved appropriate treatment for uncomplicated malaria, pneumonia symptoms and diarrhoea. Drug seller adherence to iCCM guidelines was high, without causing excessive prescription of antimicrobial medicines in this study. Further research should assess whether this effect is sustained over time and under routine supervision models.

**Electronic supplementary material:**

The online version of this article (doi:10.1186/s12936-017-2072-9) contains supplementary material, which is available to authorized users.

## Background

Uganda is one of only ten countries in sub-Saharan Africa that achieved millennium development goal 4 of a two-third reduction in the 1990 levels of under-five mortality by 2015 [1]. However, the sustainable development goals (SDG) framework has proposed a further reduction from the current 55–25 deaths per 1000 live births by 2030 [2, 3]. Acute febrile illnesses of malaria, pneumonia and diarrhoea account for 45–60% of these deaths [3, 4]. Many Ugandan febrile children either do not receive prompt healthcare or get the wrong treatment or go untreated, despite existence of cheap and effective child medicines and diagnostics [5, 6]. In household surveys, just 60% of the children suspected with uncomplicated malaria receive an anti-malarial medicine and only 23% get the recommended artemisinin-based combination therapy (ACT). Moreover, only 17% get parasitological diagnosis [7] and a meagre 14% receive ACT within 24 h from onset of fever [8]. Similarly, only 31% of under-five children who present with pneumonia symptoms receive an antibiotic, and 35% of diarrhoea cases receive oral rehydration salts (ORS), and only 2% receive zinc tablets [8].

The integrated community case management (iCCM) for paediatric febrile illnesses programme is a community level intervention where community health workers (CHWs) are trained on integrated case management of febrile illnesses. They are provided with the point-of-care diagnostics (such as antigen-based malaria rapid diagnostics tests and respiratory rate counters) and medicines (including dose-packed colour-coded ACT and dispersible tablet amoxicillin) to detect illness, classify and treat U5 children accordingly [9, 10]. CHWs are community-based volunteers, also referred to as the village health teams (VHTs) selected by village elders and peers from within the community.

Whereas studies done in rural areas in Uganda, Ethiopia and Zambia [5, 11–16] demonstrate that the iCCM intervention is an effective programme with a 40% potential reduction of U5 mortality [5], its implementation in Uganda has been fragmented and so far rolled-out to only 34 of the 112 districts [17, 18]. Additionally, iCCM implementation has been characterized by challenges of inadequate supervision, unreliable medicine and equipment supply chains, low motivation and retention of CHWs, weak monitoring and evaluation systems and low uptake of CHWs-services in certain areas [19, 20]. Consequently, poor children in remote and underserved areas in Uganda have not fully harnessed the benefits of the iCCM intervention.

To complement the unreliable, sometimes inaccessible or even non-existent facility-based services and community health programmes, people seek fever care from private drug shops and clinics [7, 21]. Drug shops are small-scale medicine outlets that are granted licenses to sell a limited list of medicines by the National Drug Authority (NDA) following successful vetting of personnel, physical premises and payment of prescribed fees [22]. The drug shops and outlets that serve remote areas have minimal health infrastructure [23] manned by people with health training from zero to 2 years [24–26]. In rural areas, up to 53% of care-seekers with U5 children first seek fever care from drug shops [6, 7, 27]. However, these drug outlets occasionally provide treatments that are inconsistent with evidence-based clinical guidelines and are potentially harmful [24, 27]. Yet, these outlets still remain the first port-of-call for poor and disadvantaged children with febrile illnesses [28].

A pilot study in Eastern Uganda infers that targeted interventions to improve drug seller treatment practice increase coverage and access to high quality care for childhood fevers in underserved high malaria endemic regions. In addition, malaria rapid diagnostic tests (RDTs) results are adhered to in prescribing anti-malarial or alternative fever treatments [29, 30]. It is not clear from this study, however, how a lower malaria transmission rate affects compliance to RDTs results and use of alternative treatments for febrile conditions [31–33]. In low malaria transmission areas, the drug seller may have to make a choice between financial incentives from dispensing antimicrobial drugs to test-negative cases and appropriate medicine use. It is also important to know if an iCCM intervention is good use of resources or not with regards to the emergent care-seeker utilization and drug seller treatment practices.

Hence, the authors set out to determine the effect of the iCCM intervention derived from the WHO/UNICEF recommended iCCM on appropriate testing and treatment for uncomplicated malaria, pneumonia symptoms and non-bloody diarrhoea among U5 children attending drug shops in two lower malaria transmission districts in South Western Uganda. In the intervention arm, drug seller adherence to the iCCM guidelines from three different data sources—care-seeker exit interviews, drug shop patient registries, and observation of drug seller–care-seeker encounters—and the trend of prescription of antimicrobials were evaluated.

## Methods

### Study setting

A prospective evaluation of the iCCM intervention in registered drug shops was done in Mbarara (intervention) and Bushenyi (comparison) districts. These districts are located approximately 250 km South West of the Uganda commercial and administrative capital [34]. They were chosen for this study because they had similar low malaria transmission rates, geographical terrain, and vegetation and a typical tropical climate with rainfall peaks in April and October during the year [35]. Recent surveillance studies estimated the malaria parasite prevalence in the region to be between 4.1% [34] and 9.3% [36]. By virtue of their location in South Western Uganda, the authors argued that the two districts were also similar on other factors that affect care-seeking from drug shops and drug seller treatment practices such as local beliefs, cultural practices, social economic status, health systems factors and medicine supply chain factors. The district drug inspector, district health educator, a cadre of CHWs and registered drug shops in both districts were available to participate.

Mbarara district had a population of 472,629 people served by 58 government health facilities, private medicine outlets and informal sector. Sheema district which is located between the two study districts served as a buffer area, minimizing the contamination of the comparison arm by the intervention. Bushenyi district had a population of 234,440 served by 36 government health facilities, private medicine outlets and numerous informal health providers [35, 37]. During the study, Bushenyi drug shops and community continued to receive the routine national malaria control and child health programme activities [18, 38, 39].

### Study design

The investigation was a quasi-experimental design with one intervention (Mbarara with iCCM in drug shops) and one comparison area (Bushenyi without iCCM in drug shops). The study was conducted for 16 uninterrupted months from June 2013 to September 2015, divided into three stages: pre-intervention, intervention and post-intervention. Quantitative data collection was done before-, during and post-intervention to assess the effect of iCCM intervention.

### Pre-intervention phase

It occurred from June to August 2013 with three major components:

#### Stakeholder mobilization and involvement

At national level, the study team consulted with the National Malaria Control Programme (NMCP) and the National Drug Authority (NDA) about implementation of the study. The approved protocol was shared with the NDA and NMCP prior to discussion and agreement on respective stakeholder roles. Subsequently, district level stakeholders including the district local government officials (Chief Administrative Officer, District Health Officer, District Health Educator, and the District Drug Inspector), pharmaceutical wholesalers, CHWs and drug sellers were consulted in inception meetings for advice on study design and extent of involvement.

#### Selection of drug shops

All registered drug shops on the list obtained from the district drug inspectors were subjected to the eligibility criteria, namely, having an up-to-date license status, location out of the municipality area (i.e. in the rural area) of the study arm, selling human medicines as opposed to veterinary medicines and providing consent to participate in the study. Only rural drug shops were eligible to participate in the study because the iCCM intervention is designed for implementation in rural and remote areas, underserved by formal health facilities, to increase adequate access to diagnostics and medicines for under-five fever management [9]. A total of 217 drug shops in the study area were assessed. In the intervention arm, out of 152 drug shops, only 61 drug shops met the inclusion criteria. The other drug shops were either located in the municipality (60) or sold veterinary medicines (31). In the comparison arm, out of 65 drug shops, 23 were selected and recruited into the study. Similarly, the other drug shops were either located in the municipality (35) or sold veterinary medicines (7). Both the drug shop owner and attendant were recruited into the study.

#### Drug shop care-seeker exit interviews

The sample size for care-seeker drug shop exit interviews was estimated using the Bennett method for cluster-sample surveys [40]. Each study drug shop was equivalent to a cluster in this study. The assumptions in this sample size estimation were: a prevalence of appropriate treatment for pneumonia symptoms of 15%, expected rate of homogeneity of proportions between clusters as compared to within clusters of 0.02 and expected responses per cluster of 26. A design effect of 1.5 was calculated. Taking this design effect, a two-sided test and level of significance of 5% gave a total of 12 clusters per study arm. However, baseline data was collected from 18 clusters in intervention and only 10 clusters in the comparison arm due to unforeseen variation in and non-performing clusters. At baseline, the number of clusters was increased from 12 to 18 in the intervention arm in order to achieve the desired sample size of exit interviews within the planned period.

Study drug shops were selected from the list provided by the district drug inspector. Directions to the first drug shop were obtained from district drug inspector. Subsequent drug shop(s) were located with directions provided by the recruited drug sellers till the sample size was achieved.

Baseline exit interviews were conducted from September 30th to November 8th 2013. Respondents in the exit interviews were care-seekers who sought care for U5 child with fever or history of fever, cough, fast breathing, difficult breathing or diarrhoea. Respondents were consecutively recruited as and when they exited the drug shop as long as they consented to participate in the study. Data was collected using a structured questionnaire by a trained interviewer, stationed at a convenient location outside the drug shop. Data on socio-demographic characteristics of the sick child and care-seeker, nature of current illness, if other health facilities were visited prior to that drug shop visit were collected. Other variables including medicines given at the drug shop, diagnostic tests conducted, time when that illness was noticed as well as reasons for seeking care at that drug shop were collected. The care-seeker exit interviews were conducted in Runyankole—the main local language used in the study area—and interviewers checked the medicines given to the care-seekers to verify responses to questionnaire items. A total of 212 and 216 care-seeker interviews were conducted in the intervention and comparison arms, respectively.

### Intervention phase

The intervention phase ran from February 2014 to September 2015. The iCCM intervention in Mbarara district consisted of four different components; namely (1) selection, training and work activities of drug sellers, (2) provision of information, education, information and communication (IEC), (3) supply mechanism by study team in partnership with pharmaceutical wholesalers in Mbarara for diagnostics (malaria RDT and respiratory rate counters) and medicines (ACT, amoxicillin dispersible tablets (DT) and zinc sulfate/ORS), (4) monthly support supervision done by study field supervisor trained in either pharmacy or clinical medicine, occasionally accompanied by the district drug inspector and district health educator. Table 1 provides details of the intervention components.

**Table 1:** Description of the different components of the integrated community case management of pediatric febrile illness (iCCM) intervention implemented in study drug shops

Intervention	Actor	Mechanism	Description	Beneficiary
Selection, training and work activities of drug sellers	Study team (study manager and field supervisor) District drug inspector District health educator	Telephone invitation of the drug sellers Using national curriculum for the integrated community case management of paediatric febrile illnesses (iCCM) intervention [9, 10], drug sellers were trained in class lectures and hands-on practical sessions 61 drug shops were supplied with iCCM treatment algorithms, patient registers, respiratory rate counters, malaria rapid diagnostic tests and child medicines	Drug sellers were trained on case detection and classification according to simple clinical signs and/or diagnostic testing of three febrile child illnesses of acute respiratory illness (ARI), malaria and diarrheal diseases The training covered signs and symptoms, danger signs, transmission, prevention, diagnostic testing and populations at risk of pneumonia, malaria and diarrhea, respectively Also, the drug sellers were trained on filling in patient registries, referral, managing drug supplies, counseling care-seekers, adverse reaction monitoring and patient follow-up for outcome	Drug sellers from 61 registered drug shops
Information, education and communication (IEC)	Study manager, study field supervisor District drug inspector District health educator	Marking of intervention arm drug shops with A2L (access to life) poster Community sensitization campaign using the MoH child health and malaria messages delivered through monthly radio talk shows by study and district staff and radio announcements CHWs attended sensitization workshops organized by study and district staff	Messages about febrile illnesses among children, importance of diagnostic testing, treatment adherence, and what to do if symptoms of the sick child persist and implementation of iCCM in drug shops were discussed in the workshop CHWs delivered these messages to households with U5 children by word-of-mouth	Drug sellers Care-seekers CHWs
Supply mechanism for medicines and diagnostics	Study manager and study field supervisor Pharmaceutical wholesalers	The project identified pharmaceutical wholesalers to supply the study medicines at subsidized prices and diagnostics at no cost to intervention arm drug shops The study purchased the pre-packaged medicines—ACTs, amoxicillin, zinc sulphate and ORS from manufacturers and provided them to pharmaceutical wholesalers in Mbarara Drug sellers presented special study medicine order forms to pharmaceutical wholesalers for re-supply Medicines were single-dose packed, color-coded for age and provided to drug shops at subsidized prices	The mRDT was a one-step, rapid, qualitative and differential test for detection of antigen—HRP-2 (histidine rich protein 2), specific for *Plasmodium falciparum* (CareStart™ from ACCESS BIO, INC. Ethiopian Branch, Yeka, Addis Ababa, Ethiopia), in finger prick blood [68] The respiratory rate counters from Moneray International Limited [69] The pre-packaged medicines included artemether-lumefantrine fixed-dose combination (from Ajanta Pharma Limited, Mumbai, India) dispersible tablets amoxicillin dispersible tablets (Amoxikid™, Kampala Pharmaceutical Industries (1996) Limited, Uganda) and for non-bloody diarrhea, combination of zinc sulphate dispersible tablets and oral rehydration salts (ORS) and artesunate suppositories for pre-referral treatment	Drug sellers
Support supervision and use of drug shop patient registry	Field supervisor trained in either clinical medicine or pharmacy District drug inspector District health educator	A field visit was conducted for every drug shop each month by field supervisor, other project staff and district health team	Intervention arm drug shops maintained a standard iCCM registry in triplicate copies where they recorded children seen, their symptoms (fever or history of fever, cough, fast or difficult breathing), diagnostic test done, the test results, treatment given and follow up action taken, respectively Copies of filled iCCM register pages were retrieved from each study drug shop monthly The study used all records of a total of 5975 children seen at the drug shops during the study period from February 2014 to September 2015	Drug sellers Pharmaceutical wholesalers

### Post-intervention phase

Towards the end of the study (May 4th to June 19th 2015), 285 and 268 drug shop care-seeker exit interviews were conducted in the intervention and comparison arms, respectively. These care-seeker exit interviews mirrored those conducted at baseline; they were conducted in drug shops that had participated in baseline exit interviews and the same structured questionnaire was used. In addition to exit interviews, trained interviewers used a pre-tested structured checklist to conduct 49 direct observations of drug seller–care-seeker interactions at intervention arm drug shops. Interviewers assessed actual verbal and nonverbal behavior of the drug sellers against the standard iCCM sick child job aid (treatment algorithm) to assess their quality of paediatric fever assessment and treatment of sick children [41].

In September 2015, the study field supervisor picked the final batch of the patient registries from all the intervention arm drug shops. At the completion of care-seeker exit interviews at respective drug shops, the field supervisor used the data collection dates and chronological order of observations to check the responses provided in care-seeker interviews against records of the same cases in the drug shop patient registry. Information of the malaria RDT results and respiratory rate was collected at this point for each interviewed care-seeker/child case.

### Outcome measures

The following outcome variables were derived as explained below.

The primary outcome variable was proportion of U5 children that received appropriate treatment (the right dose, frequency and duration for the right indication i.e. overall appropriate treatment) for each of uncomplicated malaria, pneumonia symptoms and non-bloody diarrhoea as assessed against the national treatment guidelines [10] and WHO definition of rational medicine use [42] as follows:

#### Appropriate treatment for uncomplicated malaria

A child with fever or history of fever was tested by malaria RDT, if positive received the right regimen of ACT and an afebrile child was neither tested nor prescribed ACT. Children with malaria RDT positive results should have received artemether/lumefantrine 20/120 mg DT as follows; 6 tablets in yellow pack for children aged 4–35 months (one tablet twice daily for 3 days), 12 tablets in blue pack for children aged 36–59 months (three tablets twice daily for 3 days).

#### Appropriate treatment for pneumonia symptoms

A child with cough and fast breathing (checked by respiratory timer to be 60 or more breaths per minute for a child 0–7 days, 50 or more breaths per minute for child 2–11 months and 40 or more breaths per minute for child 1–5 years) received right regimen of amoxicillin DT and child with cough and normal breathing was not prescribed amoxycillin DT. Children with cough and fast breathing should have received amoxicillin DT 125 mg as follows; 20 tablets in pink pack for children aged 2–11 months (two tablets twice daily for 5 days), 30 tablets in green pack for children aged 12–59 months (three tablets twice daily for 5 days).

#### Appropriate treatment for non-bloody diarrhoea

Child with non-bloody diarrhoea (loose stool with no visible presence of blood) received zinc 200 mg DT and ORS sachets as follows; 5 tablets for children aged 2–6 months (half tablet once a day for 10 days), 10 tablets for children aged 7–59 months (one tablet once a day for 10 days). Each of these children received two sachets of ORS and the drug seller demonstrated to care-seeker how to reconstitute. Each child was advised to drink at least half a 300 ml cup after every lose stool.

Secondary outcome variables included provision of ACT medicines, amoxicillin DT or diarrhoea treatment, as proportions of U5 febrile cases seeking treatment for fever or suspected malaria, pneumonia symptoms and diarrhoea who were sold ACT medicines, DT amoxicillin tablets or diarrhea treatment, respectively, regardless of whether or not they were examined for clinical signs (cough, fast-breathing, fever, diarrhoea) or were diagnostically tested.

The other secondary outcome variable was uptake of diagnostic tests as proportions of U5 febrile cases seeking treatment for fever or suspected malaria, pneumonia symptoms and diarrhoea that were tested with thermometer, malaria RDT or respiratory rate timer, respectively. Child cases with fever should have had their temperatures taken using a thermometer, those with fever or 24-h history of fever should have had blood sample taken off a finger-prick and tested by RDT for presence of malaria parasite antigens and children with cough should have had their respiratory rate counted with respiratory timer. Lastly, the monthly proportions of children that were prescribed any antimicrobial medicine were also derived.

The predictor variables were study (intervention) arm, pre- or post-intervention participation (time variable) of the care-seeker and child pair, interaction term between time variable and intervention. Extraneous variables including care-seeker/child characteristics and drug shop characteristics (see Table 3) were controlled for to obtain adjusted estimates of the primary outcomes.

### Statistical analysis

Quantitative data from care-seeker exit interviews was collected using tablets with Open Data Kit (ODK) software, and were cleaned, checked, coded and then transferred to Stata version 13.0 (Stata Corp., College Station, TX, USA). The outcome variables were derived prior to analysis. The encounter between drug seller and the pair of care-seeker and respective child was the unit of observation and analysis. The data was analysed in five steps. First, the care-seeker/child characteristics of the exit interview sample population in the intervention and comparison arms at baseline were compared. Categorical variables were compared by calculating cluster-weighted Chi squares, while continuous variables were compared by calculating the adjusted t value using the Stata ‘CLTEST’ modules for performing cluster-adjusted Chi square and t tests [43]. The adjustment for clustering used the intra cluster correlation estimated by large analysis of variance which applies a correction for imbalanced clusters [44]. Similar comparison was made for the end-line care-seeker exit interviews (see Table 3).

Second, the effect of the iCCM intervention on the primary outcomes was determined by estimating the net intervention effect. The Stata ‘DIFF’ module was used to determine difference between intervention and comparison arm before the iCCM intervention subtracted from the difference between the intervention and comparison arm after (also known as difference-in-difference (DiD) analysis), with respect to appropriate treatment for uncomplicated malaria, pneumonia symptoms and non-bloody diarrhoea, respectively [45]. The intervention effect(s) on the outcomes and their confidence intervals were estimated under the assumption of normally distributed residuals to facilitate presentation in percentage units. Given the small number (10–12) of clusters per study arm, bootstrapping was done in 50 replications to improve inference with clustered standard errors [46]. Selection of covariates for multivariable analysis was by backward elimination stepwise regression, after removing collinear variables from the full model. A significant level to stay in the model of 0.2 was applied. Covariates of clinical importance to the primary outcomes such as care-seeker paying for diagnostic tests and time to get from home to drug shop were forced into the model and evaluated for their effect on R-squared [47]. Thereafter, statistical significance for intervention effect was interpreted at p value less than 0.05. Similarly, DiD analysis was applied to secondary outcomes of provision of ACT, amoxicillin DT, and diarrhoea treatment, and uptake of diagnostic testing among under-five child cases presenting at the study drug shops.

Third, the primary outcomes in the intervention and comparison arms at baseline were tested for the balancing property using the “TEST” option in the Stata “DIFF” module, based on the two-sample t test [45]. Covariates with p-value at significant level to stay in the model of 0.2 were retained in the model tested for the balancing property. This balancing t test was done to show that the distribution of the primary outcome variable between the intervention and comparison arms was the same regardless of the covariates [45].

Fourth, to determine drug seller adherence to the iCCM guidelines, the authors analysed the proportions of appropriate treatment for uncomplicated malaria and pneumonia symptoms among end-line care-seeker exit interviews in the intervention arm. Also proportions of child cases in whom respiratory rate was counted, and malaria RDT done were determined, respectively. Binomial proportion exact confidence intervals were calculated. These proportions were compared with corresponding proportions derived from drug shop patient registries and direct observation of drug seller–care-seeker interactions. This allowed for triangulation of proportions across evaluation components, thereby increasing robustness of findings and providing more in-depth understanding of the observed appropriate treatment for the childhood conditions.

Fifth and last, data from drug shop registries in intervention arm were summarized into monthly proportions of child visits prescribed any antimicrobial agent and analyzed using the STATA PTREND module for trend analysis for proportions [48]. This nonparametric test calculated a Chi square statistic for trend based on regression and also analysed for departure from the trend line.

### Ethical issues

The research and ethics committees at Makerere University School of Public Health (IRB00011353), Uganda National Council of Science and Technology (HS1385) and World Health Organization approved the study. Written informed consent was obtained from drug sellers and care-seekers prior to their participation in the study.

## Results

### Sample description

All eligible drug shops in the intervention (61 drug shops) and comparison arm (23 drug shops) were recruited into the study. Table 2 shows the distribution of care-seeker–child pairs by study arm and survey.

**Table 2:** Distribution of caretaker–child pairs by study arm, survey and cluster in South Western Uganda

	Intervention arm		Comparison arm	
	Before	After	Before	After
Total number of respondents	212	285	216	268
Number of clusters	18	12	10	12
Median number of respondents per cluster	9	26	22	26
Minimum number of respondents per cluster	5	12	20	3
Maximum number of respondents per cluster	23	29	24	30

At both baseline and end-line, the care-seeker/child characteristics in intervention and comparison arms were similar except for how care-seekers decided to buy medicine (Table 3). Also, there were differences between the two arms at baseline, on reasons for seeking care at that drug shop and a higher proportion of diarrhoea cases in the intervention arm. At end-line, there were differences in distance to drug shop, being friends with the drug seller and a higher proportion of cases with rapid or difficult breathing in the intervention arm. The test for the balancing property of the intervention and comparison arms, given a set of covariates (those selected for the multivariable models by backward elimination stepwise regression—see Table 4) showed a difference in appropriate treatment for uncomplicated malaria of 23.7% (p < 0.001) in favour of the comparison arm, no difference in appropriate treatment for pneumonia symptoms between the two arms and a difference of 5.6% (p 0.548) in appropriate treatment of non-bloody diarrhoea in favour of the intervention arm.

**Table 3:** Socio-demographic characteristics of respondents seeking care at drug shops in intervention and comparison arms at baseline (2013) and end-line (2015) in South Western Uganda

Background characteristics	Baseline (2013)			End-line (2015)		
	Intervention arm (%)	Comparison arm (%)	p value	Intervention arm (%)	Comparison arm (%)	p value
	N = 212	N = 216		N = 285	N = 268	
*Care-seeker/child characteristics*						
*Categorical variables*						
Child’s symptom or sign						
Fever	128 (60.4)	111 (51.4)	0.215	172 (61.2)	112 (43.2)	0.165
Cough	133 (62.7)	148 (68.5)	0.340	191 (68.0)	148 (57.0)	0.077
Rapid or difficult breathing	44 (20.8)	42 (19.4)	0.855	133 (47.3)	26 (10.0)	< 0.001
Diarrhoea	70 (33.0)	44 (20.4)	0.030	60 (21.4)	42 (16.2)	0.266
Other symptom	58 (27.4)	43 (19.9)	0.376	91 (32.4)	71 (27.4)	0.525
Child’s sex						
Female	118 (55.7)	111 (51.4)	0.421	128 (46.4)	146 (56.8)	0.079
Care-seeker’s sex						
Female	162 (76.4)	173 (80.1)	0.558	242 (87.7)	208 (80.9)	0.364
Whether respondent had ever attended school	196 (92.5)	209 (96.8)	0.161	243 (88.0)	225 (87.6)	0.928
Highest level of school			0.164			0.843
Primary	123 (58.6)	109 (50.5)		126 (44.2)	104 (38.8)	
O-level	59 (28.1)	76 (35.2)		90 (31.6)	85 (31.7)	
A-level and higher	12 (5.7)	24 (11.1)		21 (7.4)	36 (13.4)	
None	18 (8.5)	7 (3.2)		48 (16.8)	43 (16.0)	
Respondent’s occupation			0.145			0.729
Unemployed	9 (4.2)	41 (19.0)		39 (13.7)	24 (9.0)	
Housewife	25 (11.9)	53 (24.5)		94 (33.0)	56 (20.9)	
Self employed	92 (43.8)	87 (40.3)		76 (26.7)	110 (41.0)	
Civil servant	6 (2.8)	5 (2.3)		11 (3.9)	12 (4.5)	
Other	80 (38.1)	30 (13.9)		65 (22.7)	66 (24.6)	
Perceived severity of illness			0.504			0.866
Very severe	41 (19.3)	26 (12.0)		35 (12.7)	35 (13.6)	
Moderately severe	104 (49.1)	114 (52.8)		188 (68.1)	164 (63.8)	
Not severe	67 (31.6)	76 (35.2)		53 (19.2)	58 (22.6)	
Time illness was noticed			0.086			0.627
Less than 24 h	46 (21.7)	90 (41.7)		81 (29.4)	75 (29.2)	
Between 24 and 48 h ago	75 (35.4)	82 (38.0)		105 (38.0)	125 (48.6)	
More than 48 h ago	78 (36.8)	43 (19.9)		88 (31.9)	56 (21.8)	
Do not know	13 (6.1)	1 (0.46)		2 (0.72)	1 (0.39)	
Sought care elsewhere prior to drug shop visit	59 (27.8)	52 (24.1)	0.628	53 (19.2)	57 (22.2)	0.736
How care-seeker decided to buy medicine			0.010			0.003
Knew medicine or was advised by friend	25 (26.3)	53 (38.4)		29 (27.4)	55 (44.0)	
Advised by drug seller	70 (73.7)	85 (61.6)		77 (72.6)	70 (56.0)	
*Continuous variables*						
Mean age of child (in months)	23.5 (20.0–27.0)	21.5 (17.1–25.9)	0.432	21.7 (19.2–24.2)	25.8 (23.1–28.4)	0.990
Mean caretaker age (in years)	30.3 (28.9–31.8)	28.0 (26.5–29.8)	0.034	29.3 (27.8–30.7)	30.8 (29.2–32.3)	0.939
*Drug shop characteristics*						
Reason for seeking care at drug shop						
Short distance to drug shop	70 (33.0)	138 (63.9)	0.024	132 (47.8)	193 (75.1)	0.013
Open all time	36 (17.0)	106 (49.1)	0.009	85 (30.8)	118 (45.9)	0.176
Can borrow medicine	33 (15.6)	82 (38.0)	0.019	103 (37.3)	92 (35.8)	0.881
Drug seller is my friend	37 (17.5)	69 (31.9)	0.098	156 (56.5)	96 (37.4)	0.050
Regular supply of drugs	51 (24.1)	157 (72.7)	< 0.001	92 (33.3)	128 (49.8)	0.180
Good customer service	76 (35.9)	133 (61.6)	0.041	164 (59.4)	163 (63.4)	0.730
Recommended to me	11 (5.2)	30 (13.9)	0.099	94 (34.0)	30 (11.7)	0.056
Has good or trained staff	32 (15.1)	118 (54.6)	0.008	123 (44.6)	129 (50.2)	0.667
Other	21 (10.0)	8 (3.7)	0.234	18 (6.5)	6 (2.3)	0.328
Time to get to drug shop (min)			0.012			< 0.001
< 15	67 (31.8)	94 (43.5)		95 (34.4)	124 (48.3)	
15–30	81 (38.4)	70 (32.4)		86 (31.2)	95 (37.0)	
30–60	41 (19.4)	35 (16.2)		67 (24.3)	30 (11.7)	
> 60	22 (10.4)	17 (7.9)		28 (10.1)	8 (3.1)	
If care-seeker paid for diagnostic tests	51 (19.0)	195 (90.7)	< 0.001	26 (9.4)	1 (0.4)	< 0.001
Ability to meet treatment costs	50 (89.3)	175 (92.6)	0.293	259 (93.8)	249 (96.9)	0.071

**Table 4:** Effects of the iCCM intervention on appropriate treatment for febrile childhood conditions among U5 children at drug shops in South Western Uganda from 2013 to 2014; difference-in-difference analysis

	Observed percentage (crude)		Effect estimate of the iCCM intervention (crude)			Observed percentage (adjusted)		Effect estimate of the iCCM intervention (adjusted)		
	Intervention arm	Comparison arm	Change in percentage	95% CI	p value	Intervention arm	Comparison arm	Change in percentage	95% CI	p value
Child cases with fever, pneumonia symptoms and diarrhea										
Pre-intervention	n = 212	n = 216								
Post-intervention	n = 285	n = 268								
Appropriate treatment for the childhood conditions										
Uncomplicated malaria^a^										
Pre-intervention	8.3	31.9	80.2	53.9, 106.5	< 0.001	− 65.3	− 38.5	34.5	8.6, 60.4	< 0.009
Post-intervention	57.4	0.9				− 51.9	− 59.6			
Pneumonia symptoms^b^										
Pre-intervention	0	0	65.5	51.2, 79.8	< 0.001	− 56.1	− 56.9	54.7	28.4, 81.0	< 0.001
Post-intervention	65.5	0				− 0.4	− 55.8			
Non-bloody diarrhea^c^										
Pre-intervention	51.3	45.7	31.4	0.8, 62.0	0.045	5.3	− 10.1	− 11.2	− 65.5, 43.1	0.687
Post-intervention	65.6	28.6				− 11.8	− 16.0			

^a^Controlled for child presenting with fever or history of fever, testing child with malaria RDT, counting respiratory rate of child, care-seeker being friends with drug seller, if care-seeker paid for diagnostic tests, time to get from home to drug shop, R-square = 0.75

^b^Controlled for child presenting with fever or history of fever, child presenting with cough or difficulty in breathing, testing child with malaria RDT, if child did not undergo any diagnostic testing, how care-seeker decided to buy medicine, care-seeker being friends with drug seller, care-seeker sex, child’s condition not being severe, R-square = 0.66

^c^Controlled for child presenting with signs of fever or history of fever, cough or difficulty in breathing, diarrhoea, counting respiratory rate of child, measuring temperature of child, if care-seeker was made to repeat treatment instructions, seeking care elsewhere before coming to drug shop, how care-seeker decided to buy medicine, care-seeker being able to take medicines on credit, good customer service at drug shop, R = 0.29

The largest intervention effect on appropriate treatment was recorded for uncomplicated malaria, 80.2% (95% CI 53.9, 106.5) followed by for pneumonia symptoms, 65.5% (95% CI 51.2, 79.8) and lastly for non-bloody diarrhoea, 31.4% (95% CI 0.8, 62.0). Controlling for extraneous variables (See Table 4) reduced the effect sizes as follows; appropriate treatment for uncomplicated malaria 34.5% (95% CI 8.6, 60.4), for pneumonia symptoms to 54.7% (95% CI 28.4, 81.0) and for non-bloody diarrhoea to − 11.2% (95% CI − 65.5, 43.1). Except for non-bloody diarrhoea, all percentage increases in appropriate treatment for the childhood conditions were statistically significant at p value < 0.05, even after controlling for extraneous variables (see Table 4). Also, a large negative change from 31.9 to 0.9% in appropriate treatment for uncomplicated malaria was observed in the comparison arm. Details of the proportions of child cases who presented with fever, pneumonia symptoms and non-bloody diarrhoea, proportions who were diagnostically tested and proportions who were given medicines are provided as supplementary materials (Additional file 1).

### Effect on provision of ACT, amoxicillin DT and diarrhoea treatment and uptake of diagnostic testing

The largest intervention effect was on provision of amoxicillin DT to child cases with suspected pneumonia symptoms, 91.5% (95% CI 82.5, 100.5) (Table 5) followed by provision of ACT to child cases with suspected uncomplicated malaria, 24.8% (95% CI − 3.3, 51.1) and lastly for provision of diarrhoea treatment to child cases with non-bloody diarrhoea, 17.1% (95% CI − 22.3, 53.7%). Only the percentage increase in provision of amoxicillin DT to child cases with suspected pneumonia symptoms was statistically significant at p value < 0.05.

**Table 5:** Effects of the iCCM intervention on provision of ACTs, amoxicillin DT and diarrhea treatment and uptake of diagnostic testing for febrile childhood conditions among U5 children at drug shops in South Western Uganda from 2013 to 2014; difference-in-difference analysis of data from care-seeker exit interviews

	Observed percentage		Effect estimate of the iCCM intervention		
	Intervention arm	Comparison arm	Change in percentage	95% CI	p value
Child cases with fever, pneumonia symptoms and diarrhea					
Pre-intervention	n = 212	n = 216			
Post-intervention	n = 285	n = 268			
Provision of ACT, amoxicillin DT and diarrhea treatment					
Provision of ACTs for suspected uncomplicated malaria					
Pre-intervention	46.2	32.0	24.8	− 3.3, 51.1	0.090
Post-intervention	92.6	53.6			
Provision of DT amoxicillin for suspected pneumonia symptoms					
Pre-intervention	4.8	3.1	91.5	82.5, 100.5	< 0.001
Post-intervention	93.2	0			
Provision of diarrhea treatment for non-bloody diarrhea					
Pre-intervention	65.3	49.0	17.1	− 22.3, 53.7	0.397
Post-intervention	58.0	24.6			
Uptake of diagnostic testing for uncomplicated malaria, pneumonia symptoms and fever					
Malaria RDTs					
Pre-intervention	18.5	23.6	52.6	27.3, 77.9	< 0.001
Post-intervention	47.8	0.39			
Respiratory timer					
Pre-intervention	0	0	60.1	47.6, 72.6	< 0.001
Post-intervention	60.1	0			
Thermometer					
Pre-intervention	8.5	32.4	41.2	19.4, 63.0	0.001
Post-intervention	28.3	10.9			
No investigations done					
Pre-intervention	53.6	43.5	− 53.5	− 93.9, − 13.3	0.013
Post-intervention	13.8	57.2			

The reported intervention effect on uptake of diagnostic testing from smallest to largest improvement was on use of thermometer, 41.2% (95% CI 19.4, 63.0), followed by use of malaria RDTs, 52.6% (95% CI 27.3, 77.9) and use of respiratory timers, 60.1% (95% CI 47.6, 72.6) (Table 5). There was a reduction by half (-53.5%, 95% CI − 93.9, − 13.3) of child cases who were not subjected to any diagnostic test at all. These improvements or reduction were all statistically significant.

### Drug seller adherence to iCCM guidelines in intervention arm drug shops in South Western Uganda

In the intervention arm, 285 care-seeker exit interviews were done at end-line, 5975 child cases were reported in drug shop patient registries during the intervention period from February 2014 to September 2015 and 49 drug seller–care-seeker encounters were directly observed at end-line.

**Fig. 1:**
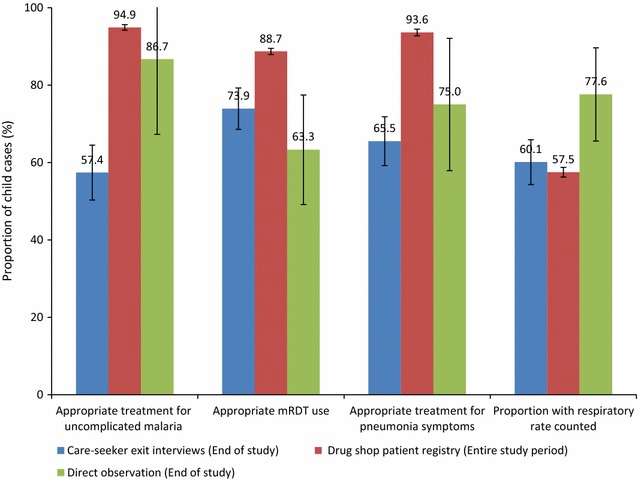
Proportion of U5 children who received appropriate treatment and diagnostic testing for pneumonia symptoms and uncomplicated malaria by three different data sources

Data from these three different sources was used to evaluate drug seller adherence to the national iCCM guidelines as presented in Fig. 1.

Generally, drug seller adherence to national iCCM guidelines was moderate for appropriate treatment of uncomplicated malaria, 57.4% (95% CI 50.3, 64.5), and pneumonia symptoms, 65.5% (95% CI 59.2, 71.8), and appropriate malaria RDT use, 73.9% (95% CI 68.6, 79.3), among the care-seeker exit interviews and highest for three outcomes among child cases reported in the drug shop patient registries, namely, appropriate treatment for uncomplicated malaria, 94.9% (95% CI 94.2, 95.6), appropriate treatment for pneumonia symptoms 93.6% (95% CI 92.8, 94.5) and appropriate malaria RDT use, 88.7% (95% CI 87.9, 89.5).

However, the proportion of child cases in whom respiratory rate was counted was lowest in drug shop patient registries, 57.5% (95% CI 56.3, 58.8), moderate among care-seeker exit interviews, 60.1% (95% CI 54.3, 65.9) and highest for direct observation 77.6% (95% CI 65.6, 89.7).

From the drug shop patient registry, 97% (3628/3738) child cases with fever had been tested for malaria using an RDT and the RDT positivity rate recorded was 47% (1957/4190) (95% CI 45–48). However, the proportion of child cases in whom treatment followed malaria RDT results was 73.9% among care-seeker exit interviews, highest (88.7%) in child cases reported in drug shop patient registries, and lowest (63.3%) in direct observation.

More details on how the drug sellers adhered with the national iCCM algorithm to detect and classify disease and treat the child cases in the intervention arm drug shops are available in the additional information (Additional file 2).

### Effect of the adapted iCCM on antimicrobial medicine use in intervention drug shops from February 2014 to September 2015

**Fig. 2:**
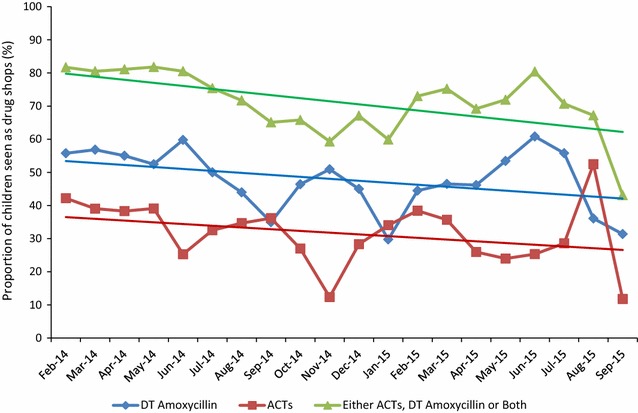
Trend of monthly proportions of children prescribed ACT medicines, amoxycillin DT or any antimicrobial medicine at study drug shops from February 2014 to September 2015

Figure 2 is a plot of monthly proportions of child cases attending the intervention arm drug shops prescribed ACTs, amoxicillin DT or at least an antimicrobial medicine (either ACT or amoxicillin DT or both) from February 2014 to September 2015. The trend analysis for proportions by regression found; i) a linear trend with negative slope (− 0.009) with p value < 0.001 for “either ACT, amoxycillin DT or both” line, and a p value 0.769 for a nonlinear trend, ii) a linear trend with negative slope (− 0.006) with p value < 0.001 and a p value of 0.587 for nonlinear trend for “Amoxycillin DT” line, and iii) a linear trend negative slope (− 0.008) with p value < 0.001 and a p value of 0.674 for nonlinear trend for “ACT” line. In summary, statistically significant linear trend lines with slight negative gradient were observed for the three scenarios.

## Discussion

The authors demonstrated that implementing an iCCM intervention at retail drug shops increased appropriate treatment for uncomplicated malaria, pneumonia symptoms and non-bloody diarrhoea, which implied that a higher proportion of U5 children received the right medicine in the right dose, frequency and duration for the right indication in the intervention arm as compared to the comparison arm in South Western Uganda.

Also, an increase in diagnostic testing for malaria, pneumonia symptoms and high temperature among U5 children with fever or history of fever or cough was observed. An increase in proportion of children prescribed recommended child medicines (ACT medicines, amoxicillin DT and diarrhoea treatment) was found, thus indicating increased availability and provision of these medicines in the intervention arm as compared to comparison arm. Overall prescription rates of antimicrobials decreased over the 20-month study period.

Findings of this study in a lower malaria endemic setting are similar to those by Awor et al. in Eastern Uganda, notwithstanding the observed higher malaria RDT positivity of 78% as compared to 47% reported in the current study [29]. Meanwhile an RDT positivity rate of 47% was higher than what was expected, given values of 5 and 9.3% malaria prevalence reported in studies conducted in the same area [34, 36]. This difference in malaria prevalence could be explained by the difference in the populations of U5 children tested, the current study tested sick children presenting at drug shops, while the 2014 malaria indicator survey [34] and the study by Kamya et al. [36] tested healthy children in their homes. However, it could also be related to possibilities of false malaria RDT positivity due to persistent HRP2 antigenaemia after malaria treatment [49], or falsification of the malaria RDT results in the patient registries by drug sellers so as to increase the sales of ACT medicines. A further study will attempt to validate the drug shop patient registry malaria RDT results.

Within the intervention arm drug shops, appropriate treatment for uncomplicated malaria was lower from care-seeker exit interviews than from drug shop patient registries and direct observation. The lower proportion observed in care-seeker interviews was likely due to increased non-adherence to malaria RDT-negative test results thereby prescribing of anti-malarial medicines to these cases. Similar findings have been reported elsewhere by Johansson et al. [50] and Burchett et al. [51], for failure to diagnose other causes of illness or to increase sale of ACT medicines.

Appropriate treatment for pneumonia symptoms was higher from drug shop patient registries than from care-seeker exit interviews and direct observation, which scored similar proportions of child cases. The higher proportion observed could be explained by over-diagnosis of pneumonia symptoms by drug sellers so as to increase antibiotic sales, or due to difficulties in applying respiratory rate cut-offs correctly, as shown by Mukanga et al. for community health workers [52].

The current study demonstrated a decline in the proportion of children prescribed either an anti-malarial or antibiotic medicine over study period of 20 months in the intervention arm. These findings contrast results of other studies that reported that testing with malaria RDTs reduced use of anti-malarial medicines but led to increased antibiotic use [53–56]. The authors believe that the integrated nature of the iCCM intervention in the current study, rather than vertical malaria intervention alone in the latter studies, contributed to this. This argument is supported by a synthesis of ten studies from multiple epidemiological and healthcare contexts by Burchett et al. That synthesis found that presence of options for alternative management of malaria negative cases promoted adherence to malaria RDT-negative results [51]. Another study reported that presence of components that modify supply side factors such as price subsidies, consistent supply at the pharmaceutical distributor points, friendly relations with regulators and demand generation activities explained the mechanism of effect [57].

However, there are notable limitations to this study. First, the intervention effect on the outcome variables was assessed in a quasi-experimental design which is amenable to multiple threats to internal validity including selection and history [58], and hence may be less compelling to infer causality. Systematic differences were observed at baseline between the intervention and comparison arms for reasons for which care-seekers sought care from the drug shops. This could be explained by drug shops in the comparison arm being closer to care-seekers’ homes and more likely to be open all the time. This was further seen in care-seekers bringing in the under-five children sooner (within 24 h) in the comparison as compared with the intervention arm. Additionally, the comparison arm (Bushenyi district) occupied a smaller land area (942.3 square kilometres) than the intervention arm (Mbarara district, 1778.4 km^2^) [59]. The authors argue that these differences do not explain the observed intervention effects. Backward elimination with stepwise regression identified only one drug shop characteristic—care-seeker being friends with drug seller—for multivariable analysis for appropriate treatment for uncomplicated malaria. And, the intervention effect remained even after controlling for the drug shop and care-seeker/child covariates. This implies the difference in appropriate treatment for uncomplicated malaria between the intervention and comparison arms is influenced by drug shop characteristics (testing child with malaria RDT, counting respiratory rate of child, care-seeker being friends with drug seller, if care-seeker paid for diagnostic tests, and time to get from home to drug shop), in addition to child presenting with fever or history of fever. The adjusted model for appropriate treatment for pneumonia symptoms showed an intervention effect while that for non-bloody diarhoea did not show any effect. Second, although the DiD analytic technique adjusts for time-invariant unobserved heterogeneity, it requires testing of the compliment of parallel paths of the outcome for the intervention and comparison arms [58]. Given the availability of only two periods, pre- and post-intervention, in this study, this assumption could not be tested. However, the balancing t-test at baseline showed that there was no difference between appropriate treatment for pneumonia symptoms and diarrhoea, respectively, even after controlling for covariates that stayed in the multivariable model at significant level of 0.2 in the backward elimination stepwise regression procedure. There was a difference in appropriate treatment for uncomplicated malaria in favour of the comparison arm at baseline. Borrowing from the “parallel growth” extension of the balancing test, as advanced by Mora and Reggio [60], the authors argue that in the absence of the intervention, this outcome variable is orthogonal to the intervention effect. Since the parallel growth assumption is flexible and allows for differing trends before and after the intervention, the difference in appropriate treatment for uncomplicated malaria observed in the DID analysis is likely due to the intervention.

Third, in the current study, the comparison arm performed better at baseline as compared to post-intervention on uptake of diagnostic testing for malaria and fever, and provision of diarrhoea treatment, (see Table 5). The observed decline from pre- to post-intervention in the comparison arm, for uptake of malaria RDTs, could be explained by substantial reduction in availability of diagnostics in the private sector pharmaceutical supply chain of Uganda caused by the closure of the affordable medicine facility for malaria (AMFm), and its subsequent transition to the Global Fund private sector co-payment mechanism (in 2014 and 2015) [61]. Similar studies have also argued that regulatory and policy barriers to implementation of malaria RDTs by drug sellers can cause problems to consistent availability of diagnostics in such retail health markets [62]. Nevertheless, appropriate malaria treatment increased also in the comparison arm. The AMFm transition, regulatory and policy barriers would not explain the current study’s findings on pneumonia and diarrhoea treatment.

Fourth, an unequal number of clusters were observed at baseline with the comparison arm registering some non-performing clusters. In addition, the clusters had unequal observations within them. However, analytical methods applied in adjusting for clustering used the intra cluster correlation estimated by large analysis of variance which applies a correction for imbalanced clusters and computes robust standard errors [44]. Fifth, the current study analysed the primary outcome at single time points before and after implementation of the adapted iCCM model in intervention arm drug shops. The observed iCCM intervention effect on assessing for and treating pneumonia symptoms could have been an over-estimation given the absence of respiratory timers and amoxycillin DT pre-intervention and in the comparison arm. However, the current study examined the uptake and utilization of these health technologies once available. This is in line with another study [63] that reported that presence of diagnostics at the drug shops was necessary but not sufficient to translate into their uptake. Other researchers [63, 64] have argued that behavioural change efforts and provider incentives are necessary. Hence, these findings infer that the iCCM intervention improved the appropriateness of drug seller treatment practices in the intervention arm as compared to the comparison arm. The strength of this study lies in the different data collection methods used and confidence in the findings is enhanced by Awor et al. [29] who observed similar improvements in quality of paediatric fever care at drug shops using multiple methods of assessing outcomes. A complete assessment of change in the trend of drug seller treatment practices would require measurements at multiple time points pre- and post-iCCM intervention and interrupted time series analysis [65–67].

Given the numerous challenges in scaling up government led iCCM in some contexts—including the lack of incentives to motivate CHWs, and national ownership of the intervention [19], drug shops could be a complementary source of healthcare going forward. However, several questions on how to construct supervision models for private sector interventions remain, as study–employed supervisors were used for the current and previous studies, rather than a scalable supervision model integrated in district health services [29]. Addressing this question is important to determine if these interventions are reasonable at scale or not. More so, Visser et al. reported that training and more frequent supportive supervision were associated with higher adherence [62].

## Conclusions

This study illustrates that implementing the iCCM intervention at retail drug shops improved appropriate treatment for uncomplicated malaria and reduced presumptive treatment of the febrile child, and promoted drug seller adherence to iCCM guidelines, also in lower malaria transmission settings of South Western Uganda. Furthermore, this study demonstrates overall declining prescription rates of anti-malarials as well as antibiotics as malaria RDTs were introduced and adhered to, contrary to other studies that introduced only malaria RDTs and led to increase in prescription of antibacterial medicines. The authors therefore argue that malaria control programmes in public as well as private sector should promote integrated case management of the sick child rather than single disease focus only. Further research should validate drug seller compliance with test results and assess the effects of the intervention in larger scale implementation, the sustainability and effect of different supervision modalities.

## Additional files

**Additional file 1.** Proportions of child cases that presented with fever, pneumonia symptoms and non-bloody diarrhoea, were diagnostically tested and were given medicines, at drug shops in South Western Uganda from 2013 to 2014, respectively.

**Additional file 2.** Table showing proportions: classification and treatment of children attending drug shops in Intervention arm in South Western Uganda from February 2014 to September 2015; comparison of different data sources.

## Abbreviations

ACT: artemisinin-based combination therapy
A2L: access to life
AMFm: affordable medicine facility for malaria
CHWs: community health workers
DiD analysis: difference-in-difference analysis
DT: dispersible tablets
HRP2: histidine rich protein 2 antigen
IEC: information, education and communication
iCCM: integrated community case management
RDT: rapid diagnostic test
MoH: Uganda Ministry of Health
NDA: National Drug Authority
NMCP: National Malaria Control Programme
ORS: oral rehydration salts
SDG: sustainable development goals
U5: under-five
UNICEF: United Nations Children’s Fund
VHTs: village health teams
WHO: World Health Organization
