# Supplementary material for: Integrated community case management by drug sellers influences appropriate treatment of paediatric febrile illness in South Western Uganda: a quasi-experimental study
Source: Malar J. 2017 Oct 23;16:425. doi: 10.1186/s12936-017-2072-9 (PMC5654057; doi:10.1186/s12936-017-2072-9)
Supplement: Supplementary file 1 — Additional file 1. Proportions of child cases that presented with fever, pneumonia symptoms and non-bloody diarrhoea, were diagnostically tested and were given medicines, at drug shops in South Western Uganda from 2013 to 2014, respectively. [file 12936_2017_2072_MOESM1_ESM.docx]

**Table S1 Proportions of child cases that presented with fever, pneumonia symptoms and non-bloody diarrhoea, were diagnostically tested and were given medicines, respectively**

| **Characteristic** | **Intervention arm** | | **Comparison arm** | |
| --- | --- | --- | --- | --- |
|  | **Before** | **After** | **Before** | **After** |
|  | **Proportion (%)** | **Proportion (%)** | **Proportion (%)** | **Proportion (%)** |
| **Fever or suspected malaria** |  |  |  |  |
| Proportion presenting with fever or history of fever | 128/212 (60.4) | 172/281 (61.2) | 111/216 (51.4) | 112/259 (43.2) |
| Proportions tested with malaria RDT | 39/211 (18.5) | 132/276 (47.8) | 51/216 (23.6) | 1/257 (0.4) |
| Proportion given any antimalarials | 93/211 (44.1) | 68/276 (24.6) | 75/216 (34.7) | 69/257 (26.8) |
| *Proportion given ACT medicines* | *43/93 (46.2)* | *63/68 (92.7)* | *24/75 (32.0)* | *37/69 (53.6)* |
| *Proportion with correct ACT dose, frequency and duration* | *9/43 (20.9)* | *54/63 (85.7)* | *2/24 (8.3)* | *27/37 (73.0)* |
| Proportion appropriately treated for uncomplicated malaria | 11/133 (8.3) | 108/188 (57.5) | 38/119 (31.9) | 1/112 (0.9) |
| **Cough or difficulty in breathing (pneumonia symptoms)** |  |  |  |  |
| Proportion presenting with cough or difficulty in breathing | 139/212 (65.6) | 200/285 (70.2) | 154/216 (71.3) | 149/268 (55.6) |
| Proportion in whom respiratory rate was counted | 0 | 166/276 (60.1) | 0 | 0 |
| Proportion given any antibiotics | 84/211 (39.8) | 160/276 (58.0) | 96/216 (44.4) | 67/257 (26.1) |
| Proportion given Amoxicillin DT | 4/84 (4.8) | 149/160 (93.1) | 3/96 (3.1) | 0 |
| Proportion with correct Amoxicillin DT dose, frequency and duration | 0 | 144/149 (96.6) | 1/3 (33.3) | 0 |
| Proportion appropriately treated for pneumonia symptoms | 0 | 144/220 (65.5) | 0 | 0 |
| **Non-bloody diarrhoea** |  |  |  |  |
| Proportion presenting with non-bloody diarrhoea | 70/212 (33.0) | 60/281 (21.4) | 44/216 (20.4) | 42/259 (16.2) |
| Proportion given any treatments for diarrhoea | 72/212 (34.0) | 69/281 (24.6) | 49/216 (22.7) | 57/259 (22.0) |
| *Proportion given Zinc tablets and ORS* | *47/72 (65.3)* | *40/69 (58.0)* | *24/49 (49.0)* | *14/57 (24.6)* |
| *Proportion appropriately treated for non-bloody diarrhoea* | *40/78 (51.3)* | *40/69 (58.0)* | *21/49 (42.9)* | *12/57 (21.1)* |
