# Supplementary material for: Integrated community case management by drug sellers influences appropriate treatment of paediatric febrile illness in South Western Uganda: a quasi-experimental study
Source: Malar J. 2017 Oct 23;16:425. doi: 10.1186/s12936-017-2072-9 (PMC5654057; doi:10.1186/s12936-017-2072-9)
Supplement: Supplementary file 2 — Additional file 2. Table showing proportions: classification and treatment of children attending drug shops in Intervention arm in South Western Uganda from February 2014 to September 2015; comparison of different data sources. [file 12936_2017_2072_MOESM2_ESM.docx]

**Table S2 Classification and treatment of children attending drug shops in Intervention arm in South Western Uganda from February 2014 to September 2015; comparison of different data sources.**

| **Characteristic** | **Care-seeker exit interviews** | | **Drug shop patient registry** | | **Direct observation** | |
| --- | --- | --- | --- | --- | --- | --- |
|  | *After* | | *Entire study period* | | *After* | |
|  | **Proportion (%)** | **95% CI** | **Proportion (%)** | **95% CI** | **Proportion (%)** | **95% CI** |
| Proportion of children with fever | 172/281 (61.2%) | 55.3 – 66.8 | 3738/5975 (62.6%) | 61.3-63.8 | 34/49 (69.4%) | 54.7 – 80.9 |
| Fever and tested with malaria RDT | 116/172 (67.4%) | 59.9 – 74.4 | 3628/3738 (97.1%) | 96.5-97.6 | 20/34 (58.8%) | 40.7 – 75.4 |
| Malaria RDT positivity | - | - | 1957/4190 (46.7%) | 45.2-48.2 | - | - |
| Appropriate malaria RDT use ^a^ | 204/276 (73.9%) | 68.3 – 79.0 | 5302/5975 (88.7%) | 87.9-89.5 | 31/49 (63.3%) | 48.3 – 76.6 |
| RDT positive and prescribed ACT | - | - | 1840/1957 (94.0%) | 92.9-95.0 | - | - |
| Appropriate treatment for uncomplicated malaria ^c^ | 108/188 (57.4%) | 50.2 – 64.4 | 3445/3628 (94.9%) | 94.2-95.6 | 13/15 (86.7%) | 59.5 – 98.3 |
| Proportion with cough or difficulty in breathing | 200/281 (71.2%) | 65.5 – 76.4 | - | - | 38/49 (77.6%) | 63.3 – 87.4 |
| Proportion with respiratory rate counted | 166/276 (60.1%) | 54.2 – 65.8 | 3446/5975 (57.5%) | 56.4-58.9 | 38/49 (77.6%) | 63.3 – 87.4 |
| Proportion with pneumonia symptoms | - | - | 2807/5975 (47.0%) | 45.7-48.3 | - | - |
| Pneumonia and amoxicillin dispensed | - | - | 2666/2807 (95.0%) | 94.1-95.8 | - | - |
| Appropriate treatment for pneumonia symptoms ^f^ | 144/200 (65.5) | 58.9 – 71.5 | 3191/3411 (93.6%) | 92.7-94.4 | 21/28 (75.0%) | 55.1 – 89.3 |
| ^a^ Appropriate malaria RDT use defined as proportion of febrile cases tested and afebrile cases not tested with malaria RDTs divided by total number of children seen at drug shop.  ^c^ Appropriate malaria treatment defined as proportion of malaria RDT positive cases prescribed recommended ACT medicines and malaria RDT negative cases not prescribed any antimalarial medicine divided by total number of febrile children.  ^f^ Appropriate pneumonia treatment was defined as proportion of child cases with fast breathing pneumonia prescribed recommended antibiotics and child cases with cough and normal breathing not prescribed any antibiotic medicine divided by total number of child cases whose respiratory rate was counted. | | | | | | |
